# High dose prolonged treatment with nitazoxanide is not effective for cryptosporidiosis in HIV positive Zambian children: a randomised controlled trial

**DOI:** 10.1186/1471-2334-9-195

**Published:** 2009-12-02

**Authors:** Beatrice Amadi, Mwiya Mwiya, Sandie Sianongo, Lara Payne, Angela Watuka, Max Katubulushi, Paul Kelly

**Affiliations:** 1 Tropical Gastroenterology and Nutrition Group, University of Zambia School of Medicine, Lusaka, Zambia; 2 Institute of Cell and Molecular Science, Barts & The London School of Medicine, Queen Mary University of London, London, UK

## Abstract

**Background:**

Treatment of cryptosporidiosis in HIV infected children has proved difficult and unsatisfactory with no drugs having demonstrable efficacy in controlled trials except nitazoxanide. We hypothesised that a prolonged course of treatment with high dose nitazoxanide would be effective in treating cryptosporidiosis in HIV positive Zambian children.

**Methods:**

We performed a double-blind, randomised, placebo controlled trial in paediatric patients in the UTH in Lusaka. The study included HIV positive children between one and eleven years of age if 2 out of 3 stool samples were positive for oocysts of *Cryptosporidium *spp. Children were given nitazoxanide suspension in a dose of 200 mg twice daily (bid) for 28 days (if 1-3 years old) or 400 mg bid for 28 days (if 4-11 years old), or matching placebo.

**Results:**

Sixty children were randomised and 52 were fully evaluated. Only five children were 4 years of age or over and received the higher dose. In the primary efficacy analysis, 11 out of 26 (42%) in the active treatment group achieved a 'Well' clinical response compared to 8 out of 26 (35%) in the placebo group. Parasitological response was declared as 'Eradicated' in 27% in the active group and 35% in the placebo group. Mortality (16/52, 31%) did not differ by treatment allocation.

**Conclusion:**

We found no significant benefit in children with cryptosporidiosis despite high dose and longer treatment duration. This is the second randomised controlled trial to suggest that in Zambian children with HIV-related immunosuppression nitazoxanide does not eradicate this infection nor provide clinical symptom reduction.

**Trial Registration:**

The trial was registered as ISRCTN41089957.

## Background

Cryptosporidiosis is an important cause of morbidity and mortality in malnourished children [1,2] and in children with AIDS [2]. In immunocompetent children cryptosporidiosis is a frequent cause of diarrhoea [3], and even these episodes which are usually of limited duration can have long-term consequences [4]. However, treatment remains difficult and unsatisfactory, with no drugs having any proven efficacy except nitazoxanide [5-7]. We have previously reported that three day treatment with the anti-parasitic drug nitazoxanide was effective in treatment of HIV-seronegative children but demonstrated insignificant activity in HIV-seropositive children [8]. There is an urgent need for effective treatment for cryptosporidiosis in HIV-infected children [9]. We postulated that the performance of this drug in such patients could be improved by a higher dosage and by giving a longer course of treatment. We report here the results of a randomised controlled trial to evaluate this hypothesis in the same setting as our earlier study. The doses of nitazoxanide chosen were double those used in the previous study, and the duration was increased from 3 to 14 days. Ethics approval was obtained from the University of Zambia Research Ethics Committee. The trial was registered as ISRCTN41089957.

## Methods

We carried out a double-blind, randomised, placebo controlled trial in paediatric patients shown to be positive for both HIV and cryptosporidiosis, in the children's diarrhoea/malnutrition ward of the University Teaching Hospital, Lusaka. Children were included if between 1 and 11 years of age, if positive in at least 2 of 3 stool samples for *Cryptosporidium *spp. (oocysts identified using auramine phenol staining), if they had had diarrhoea with 3 or more unformed stools daily, and if HIV seropositive as confirmed by the Capillus Rapid Test (Trinity Biotech, Ireland). If *Cryptosporidium *spp. oocysts were present in the initial screening but not in the baseline samples, the patient was excluded unless they tested positive for oocysts within one week from this first sample. Children were excluded if they had a bacterial cause for diarrhoea or if positive for *Entamoeba histolytica *or *Giardia lamblia *by the Triage^® ^Parasitic Panel (Biosite Diagnostics, San Diego, CA). Other exclusion criteria were a history of investigational drug therapy within one month prior to enrolment, or other recognised anti-protozoal therapy within two weeks prior to enrolment. Children who were moribund were not randomised and gravely ill were observed for a period of 1-2 weeks prior to randomisation.

### Study procedures

At the initial visit a complete medical history was reviewed to evaluate conformity with inclusion and exclusion criteria. A physical examination was conducted including body weight, height and mid-upper arm circumference. Clinical symptoms and a subjective assessment of stool features (consistency, frequency, presence of blood of mucus) were recorded. Blood tests were carried out including complete blood count, alanine aminotransferase, aspartate aminotransferase, γ-glutamyl transferase, alkaline phosphatase, creatinine, random blood glucose, electrolytes (Na and K) and a CD4 count. Urinalysis was also carried out. Blood and urine tests were also performed at week 2 (day 15) and the end of the treatment, and the physical examination was repeated on days 8, 15, 22 and the end of the study (day 28). Case report forms were used to record any adverse affects of the drug.

### Randomisation and masking of treatment allocation

Eligible candidates were randomly assigned a sequential patient number; using this number a medication pack of either placebo or nitazoxanide was dispensed. This corresponded to a computer-generated random number list generated by the study sponsor, the Romark Institute for Medical Research, Tampa, Florida, USA. The randomisation code was generated in blocks of 10 so that active and placebo groups balanced in each sequential group of 10. Children were given nitazoxanide suspension in a dose of 200 mg twice daily (bid) for 28 days (if 1-3 years old) or 400 mg bid for 28 days (if 4-11 years old), or matching placebo. This represents an average dose of 54 mg/kg/day. Only five children received the higher dose. The dose was administered as 10 ml of a suspension containing 20 mg/ml nitazoxanide or placebo (for younger children), and 20 ml of this suspension for the older children. Both nitazoxanide and placebo were administered in powder form and reconstituted into a strawberry flavoured paediatric suspension every morning and evening; nitaxozanide and placebo suspensions were identical in appearance, odour and taste. Recruitment decisions were made by the study physician (BA) and nurse leader (AW) and treatment allocation initiated by them using the numbered containers. The nitazoxanide was supplied by Romark Laboratories in containers identifiable only by a code on the label, the key to which was held by Romark Laboratories until the end of the trial so that the trial was fully double-blind. All participants of the trial remained in hospital for the duration of the treatment and had access to intravenous fluids, antibiotics, oral rehydration therapy and micronutrients (including zinc and vitamin A). Nutritional support in the form of skimmed milk based feed was provided throughout the study.

A provision was made for the supply of open label treatment with nitazoxanide for patients experiencing clinical deterioration throughout the trial or in the event of non-response after the initial treatment phase of the study. This was to reduce the possible disadvantages of being in the placebo group. If patients initially responded to treatment but relapsed or were re-infected during the 60 days following treatment open label treatment was also available. This was also an option to children excluded from the trial. Patients receiving open label treatment were monitored regularly and blood, urine and faecal samples were analysed using trial protocols.

### Clinical and parasitological endpoints

While in hospital study subjects were administered test medication daily every 12 hours and clinical symptoms and any possible adverse effects were assessed and documented daily. Clinical response was recorded as 'Well' if there were no symptoms of *Cryptosporidium *spp. infection and no watery stools in the 48 hours leading up to the final dose of nitazoxanide. If there was clinical deterioration or worsening symptoms after at least 24 hours of treatment the subject was considered to show 'Clinical Treatment Failure' and open-label rescue therapy would be implemented. The status of 'Continuing Illness' was used to classify patients without a 'Well' or 'Clinical Treatment Failure' response. Stool samples were examined every Monday, Wednesday and Friday until two consecutive samples were judged to be clear. Auramine phenol staining technique was used to identify *Cryptosporidium *spp. oocysts in concentrated and unconcentrated stools. Parasitologic response was either identified as 'Eradication' when no oocysts of *Cryptosporidium *spp. were observed in two consecutive stool samples or 'Persistence' for any outcome other than 'Eradication'. Clinical responses were declared after joint consultation by the study team physician (BA) and lead nurse (AW) and the parasitological response was declared by the team parasitologist (SS).

### Data analysis

A modified intent-to-treat population was used for the primary efficacy analysis, as specified in the protocol. This population included all patients randomised excluding (1) patients who did not receive any study medication, (2) patients with no oocysts in the baseline stool sample if *Cryptosporidium *infection was not confirmed by one of the two stool examinations within one week of baseline, and (3) patients with bacterial causes of diarrhoea identified by stool culture. The primary endpoints were the proportion of children achieving a well clinical response and time to a well clinical response. Secondary endpoints were: eradication of *Cryptosporidium *spp. oocysts from stool and time to eradication, a combination of time to eradication of oocysts and well clinical response, and mortality rates. A further secondary endpoint was the rate of improvement in diarrhoea frequency during treatment as determined by subject-specific regression modelling [10].

Time to well clinical response was calculated from the interval between the date of first administration of nitazoxanide and the date of 'Well' clinical response recorded by physician. The time to eradication of oocysts from stool was calculated from the interval between the date of first administration of nitazoxanide and the date of the first consecutive negative examination for oocysts. For the secondary endpoint of clinical response and eradication, this is regarded for each patient as the longer of the two from either time to eradication of oocyst or time to clinical response. Adverse events were coded in accordance with the COSTART dictionary and proportions of patients experiencing these affects were compared with Fisher's exact test. Laboratory tests were compared by treatment group using analysis of variance tests for comparing means and chi-square tests to compare proportions. Statistical analysis was carried out using Stata 10 (Stata Corp, College Station, Texas).

The sample size was calculated from previous experience where there was 80% clinical response rate for the active treatment group and a 35% response rate for the placebo treatment group after two weeks of treatment. Sixty children who were both HIV seropositive and tested positive for *Cryptosporidium *spp. were included in the trial. With a sample size in each group of 25 the trial was predicted to have 80% power with 95% confidence to detect the difference sought between the two groups. There was no provision for an interim analysis or for stopping the trial.

## Results

**Figure 1:**
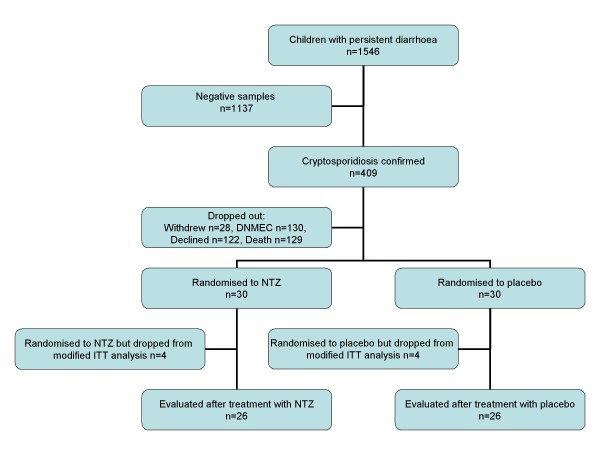
**Flow of participants through the trial**.

Of 409 children with cryptosporidiosis identified during the duration of the trial, 130 did not meet the entry criteria, 122 declined (mostly due to reluctance of the family to consent to hospitalisation for the period of the study), 129 seriously ill children died during the period of observation and 28 withdrew before randomisation (Figure 1). Sixty HIV seropositive children with cryptosporidiosis were randomised during the period of the trial which was from October 2002 to June 2004. Eight subjects (four from each treatment group) were excluded from the modified intent-to-treat population because they were not still passing oocysts at baseline (n = 4) or because stool culture identified bacterial causes of diarrhoea (n = 5). One subject was excluded for both of these reasons. The demographic characteristics of the children enrolled are shown in Table 1.

**Table 1:** Baseline characteristics of trial participants

	Intention-to-treat (ITT) analysis		Modified ITT analysis	
	**NTZ**	**Placebo**	**NTZ**	**Placebo**

Number of children	30	30	26	26

Sex (M:F)	14:16	20:10	13:13	18:8

Age (months)	20 (11.3)	21 (20.2)	21 (11.9)	22 (17.8)

CD4 count (cells/μL)	579 (378)	614 (403)	583 (316)	503 (394)

Weight (kg) (mean, SD)	7.2 (1.8)	7.3 (2.0)	7.3 (2.0)	7.3 (2.0)

Presence of oedema (number, %)	6 (20)	5 (17)	5(19)	4 (15)

Hb (g/dL) (mean, SD) [range]	8.7 (2.2) [4.9-12.8]	8.3 (1.8) [4.5-13.1]	8.3 (1.8) [5.8-11.9]	8.2 (2.0) [4.5-13.1]

WBC (×10^9^/L)	11.6 (6.2) [4.7-35]	11.0 (5.1) [3.2-21.9]	12.5 (6.7) [5.2-35]	10.9 (5.1) [4.7-13.1]

Na^+ ^(mmol/L)	137.2 (2.5)	137.6 (1.9)	137.3 (3.3)	138.3 (1.0)

K^+ ^(mmol/L)	4.4 (0.5)	4.4 (0.5)	4.5 (0.6)	4.5 (0.5)

Creatinine (μmol/L)	95.5 (39.2)	89.1 (38.3)	95.6 (34.8)	92.4 (38.2)

Alkaline phosphatase (i.u./l)	148 (72)	164 (69)	151 (77)	168 (61)

AST (i.u./l)	62.1 (61)	61 (22)	54 (23)	64 (22)

ALT (i.u./l)	45 (30)	48 (25)	45 (30)	48 (26)

Values shown are mean (SD) unless otherwise specified; where shown, values in square brackets represent range. Statistical testing using the t test or Fisher's exact test showed no significant differences between NTZ and placebo groups by either ITT or PP analysis.

### Primary endpoints

The primary efficacy analysis (Table 2) showed 11 out of 26 subjects (42%) in the active treatment group showed a 'Well' clinical response compared to 8 out of 26 (31%) in the placebo group (*P *= 0.39). In an intention-to-treat analysis, time to 'Well' clinical response was (mean, SD) 12.2 (9.6) days in the NTZ group and 13.8 (9.7) days in the placebo group (P = 0.52 using *t *test). These figures were also compared using a log-rank test and no significant difference was observed (*P *= 0.37).

**Table 2:** Clinical and parasitological responses

	Intention-to-treat analysis		Modified ITT analysis	
	**NTZ**	**Placebo**	**NTZ**	**Placebo**

Number of children	30	30	26	26

*Primary endpoints*				

Proportion achieving well clinical response (number, %)	14 (47)	12 (40)	11 (42)	8 (31)

Time to well clinical response(days)	11.4 (7.2)	7.9 (4.8)	12.2 (9.6)	13.8 (9.7)

*Secondary endpoints*				

Parasitological eradication (number, %)	10 (33)	12 (40)	7 (27)	9 (35)

Time to parasite eradication (days)	8.7 (7.0)	11.7 (9.0)	8.0 (6.3)	13.8 (9.5)

Time to combined clinical and parasitological response	19.1 (11.5)	20.3 (10.1)	12.7 (8.4)	14.2 (7.8)

Mortality (deaths by 4 weeks)	11 (37)	6 (20)	10 (38)	6 (23)

Rate of reduction in stool frequency (expressed as change per day during treatment) (mean, SD)	-0.21 (0.34)	-0.16 (0.25)	-	-

Nutritional response (change in weight over 4 weeks, kg) (mean, SD)	0.8 (1.0)	1.2 (0.9)	1.3 (0.8)	1.6 (0.8)

- not analysed; ITT, intention-to-treat. Rate of change in diarrhoea frequency was only analysed on a strict ITT basis. Statistical testing using the *t *test or Fisher's exact test showed no significant differences between NTZ and placebo groups.

### Secondary endpoints

Parasitological response (Table 2) was declared as 'Eradicated' in 7 out of 26 subjects (27%) in the active group compared to 9 out of 26 (35%) in the placebo group (*P *= 0.55). Time to 'Eradicated' parasitological response was (mean, SD) 15.3 (11.3) days in the NTZ group and 16.1 (10.6) days in the placebo group (P = 0.79 using *t *test). These figures were also compared using a log-rank test and no significant difference was observed (*P *= 0.65). Combined clinical and parasitological response was observed in 5 out of 26 (19%) in the nitazoxanide group showed the combination of these features compared with 4 out of 26 (15%) in the placebo group (P = 0.71 using *t *test). There was no significant difference in the time for these features between the two test groups (*P *= 0.68 by the log-rank test). When trends in diarrhoea frequency were compared using subject-specific regression modelling, no difference was found in the regression (β-) coefficients between the two groups (*P *= 0.55).

### Mortality

Ten out of twenty six patients (38%) in the active treatment group died during the course of the study compared to six out of twenty six patients (23%) in the placebo treatment group (P = 0.23 by χ^2 ^test) although there was no significant difference in terms of survival time by treatment group (*P *= 0.34, log-rank test).

### Adverse Events

56 adverse events were recorded in 21 different patients, 13 of these were in the nitazoxanide group and 8 were in the placebo. Of these events, 52 were considered to be serious although all of these were related to the patients' HIV-related illnesses and were considered to be not related to the treatment. Two events were considered to be possibly related to the treatment and these were one case of moderate vomiting, and a case of mild discoloration of the sclera (liver function tests normal) both observed in the nitazoxanide treatment group. The proportions of patients reporting adverse events were compared by group and there were no significant differences in the frequency or nature of adverse events reported in the two treatment groups.

## Discussion

Cryptosporidiosis is a serious infection which carries increased mortality in children in tropical countries. The severity of this mortality problem is evident from the deaths in this trial despite attentive medical and nursing care, and this underscores the need for more effective treatments. In a previous randomised, double-blind, placebo controlled trial, nitazoxanide was an effective treatment for HIV negative children with infection with *Cryptosporidium *spp. and even at a lower dosage resulted in resolution of the diarrhoea in only three days [8]. In that trial we were unable to demonstrate any significant benefit from the use of higher dose and prolonged treatment with nitazoxanide in HIV positive patients with cryptosporidiosis. In the current study we were again not able to demonstrate any effect of nitazoxanide in this group of patients, even at higher dose. The situation remains that in immunocompromised patients there is still no effective treatment for this important infection [6,7,9] even though non-randomised evidence suggests that in individual cases there may sometimes be a response [11].

It appears that in the face of HIV-related immunosuppression even a powerful antiprotozoal agent such as nitazoxanide [12,13] cannot eradicate *Cryptosporidium *spp. The precise mechanism of this attenuation of anti-parasitic drug effect is not clear but it would be expected to be related to a decline in population of CD4 (probably Th1-mediated) cells in the gut [14-16]. It is also possible that it could be a specific relative deficiency of interferon-γ. It would be interesting to compare children with varying degrees and causes of immunosuppression who also have cryptosporidiosis to identify the particular mechanism of evasion employed by the protozoa during persistent infection. There could also be an effect of varying parasitic load among trial candidates. It has long been suspected that different individuals suffer from different degrees of parasitaemia due to innate, possibly genetic, factors and it may be that both recovery from diarrhoea and drug efficacy depend on the initial load of parasites present. It is also evident that any further trials of nitazoxanide would benefit by greater understanding of the mechanisms involved in suppression of *Cryptosporidium *spp. and hopes for treatment of this protozoan pathogen would be enhanced by an improved understanding of the immune responses that can clear it in immunocompetent individuals.

This study was designed to outline any possible benefit of treating children suffering from AIDS related cryptosporidiosis with an increased dosage of nitazoxanide. Previous trials had used lower dosages and a limited treatment period showing no substantial improvement. The sample size was set to detect only a large effect of nitazoxanide as we believe that only large treatment effects are of real interest in a very vulnerable group of patients, such as this, in a resource-poor setting. This may reduce the power of the study. Our results show no significant benefit in this increased dosage and longer course of treatment for these patients. Although it has been previously shown in adults with AIDS related cryptosporidiosis that nitazoxanide can improve to a modest extent the clearance of the parasite and reduce the period of clinical symptoms [10], we saw no such effect here. As anticipated in this group of children [2], mortality was high (28% within 28 days). This may have reduced the power of the study to detect efficacy as only 18 in the nitazoxanide group and 19 in the placebo group actually completed the course of treatment. We carried out a modified intention-to-treat analysis as the trial protocol specified that children without oocysts at randomisation would be excluded from the analysis, as would children with bacterial causes of diarrhoea. Nevertheless, there was little suggestion of efficacy against any of several endpoints no matter what sort of analysis was conducted.

## Conclusion

At this dose and duration of treatment, nitazoxanide cannot be recommended for HIV-related cryptosporidiosis in Zambian children.

## List of Abbreviations

HIV: human immunodeficiency virus; UTH: University Teaching Hospital, CD4: cluster differentiation antigen 4.

## Competing interests

The authors declare that they have no competing interests.

## Authors' contributions

BA, MM and PK designed the study; BA, MM, SS, AW and PK contributed to data collection; BA, LP, MK and PK analysed the data; BA, MK, LP and PK wrote the first draft of the manuscript, and all authors reviewed the manuscript during preparation and approved the final manuscript.

## Pre-publication history

The pre-publication history for this paper can be accessed here:

http://www.biomedcentral.com/1471-2334/9/195/prepub
